# Case report: safety and efficacy of lidocaine infusion for the treatment of intractable zoster-associated neuralgia in solid organ transplant recipients

**DOI:** 10.3389/fphar.2024.1422778

**Published:** 2024-07-18

**Authors:** Huan Zheng, Bixin Zheng

**Affiliations:** ^1^ Department of Anesthesiology, Sichuan Provincial People’s Hospital, Sichuan Academy of Medical Sciences, School of Medicine, University of Electronic Science and Technology of China, Chengdu, China; ^2^ Department of Pain Management, West China Hospital, Sichuan University, Chengdu, China

**Keywords:** herpes zoster, post-herpetic neuralgia, neuropathic pain, solid organ transplantation, intravenous, lidocaine, case series

## Abstract

**Introduction:**

Solid organ transplant recipients are at high risk for developing severe zoster-associated neuralgia, and the pharmaceutic therapies of pain management for these patients with limited organ function are challenging. Intravenous lidocaine infusion showed positive analgesic effects and is used for the management of neuropathic pain. This case series reports the safety and effectiveness of intravenous lidocaine infusion in the treatment of intractable zoster-associated neuralgia in solid organ transplant recipients.

**Case series presentation:**

Five solid organ transplant recipients suffering from refractory zoster-associated neuralgia (numeric rating scale 8–10, despite using high doses of antiepileptic drugs or combined with opioids) were enrolled. Intravenous lidocaine (5 mg/kg ideal bodyweight) was administered over 1.5 h with the monitoring of vital signs. Pain intensity, patient satisfaction, adverse events, typical liver, and kidney function were evaluated. All subjects reported high satisfaction with their treatment and effective pain relief at the 6-month follow-up. One patient experienced short and mild numbness in the mouth and dizziness after the therapy, but no major adverse reactions were reported.

**Conclusion:**

This case series provides evidence that intravenous lidocaine infusion provided effective pain relief as an analgesic treatment option for transplant patients with intractable zoster-associated neuralgia.

## Introduction

Varicella zoster virus (VZV) is a highly contagious and globally distributed human pathogen that causes chickenpox (varicella) as a primary infection, usually in children belonging to areas where vaccination is not practiced. In younger adults and children, the rash and pain are generally less severe (Chen et al., 2024). After primary infection, the virus establishes lifelong latency in the sensory ganglia. When the immune system is suppressed, reactivation of VZV occurs and leads to herpes zoster (HZ), causing acute or chronic pain known as zoster-associated neuralgia. Direct viral damage, immune response-induced inflammation, and demyelination within the cranial or spinal nerve are the underlying causes of neuropathic pain (Tang et al., 2023). In older adults, typically over the age of 50, it is more severe and is often associated with post-herpetic neuralgia (PHN), causing refractory, long-lasting neuropathic pain that can severely affect a patient’s quality of life, physical functioning, and ability to perform daily tasks (Bricout et al., 2015; Florea et al., 2024).

Transplantation is the only curative therapeutic option for terminal organ failure. Due to the need for lifetime continuous immunosuppressive therapies, the incidence of HZ has been reported to be two to five fold in transplant recipients compared with the general population belonging to all age groups (Pergam et al., 2019; McKay et al., 2020). Moreover, the severity of neuralgia is also higher in these patients, which may increase the morbidity and mortality (Kwon et al., 2021). However, the pharmaceutic therapies for pain management in these patients with limited organ function are challenging.

Lidocaine is an amide local anesthetic, acting predominantly through blockade of sodium channels in the neuronal cell membrane, thereby reducing input from nociceptors (Foo et al., 2021). Intravenous lidocaine infusion is increasingly used as part of multimodal analgesic treatment for intractable neuropathic pain, which has additional sedative and anti-inflammatory properties with minimal side effects (Przeklasa-Muszynska et al., 2016; Tully et al., 2020). In this study, we first report the safety and effectiveness of intravenous lidocaine infusion on solid organ transplant (SOT) recipients with PHN.

## Case series

### Case series design

After obtaining approval from the IRB at West China Hospital of Sichuan University, a retrospective case series analysis was conducted. Inclusion criteria were SOT recipients with a diagnosis of HZ or PHN and a pain score of 4 or greater on an 11-point Numeric Rating Scale (NRS) without satisfactory pain relief from drug treatment (e.g., antidepressants, anticonvulsants, and opioids) or interventional procedures (such as nerve block, radiofrequency, and spinal cord stimulation). Patients were excluded (1) if they had a concomitant pain syndrome that could affect the pain evaluation; (2) any history of cardiac arrhythmias or taking antiarrhythmic drugs; (3) a resting heart rate of less than 50 beats/min on electrocardiogram (ECG) screening; (4) allergies to lidocaine or other local anesthetics. This case report meets applicable EQUATOR (Enhancing the Quality and Transparency Of health Research) network CARE guidelines.

### Intervention

The intravenous lidocaine infusion protocol was according to our previous study (Liu et al., 2018). Before infusion, 3 mg granisetron was used to prevent nausea and vomiting. The patient was monitored via a sphygmomanometer, pulse oximeter, and three-lead EEG, and intravenous lidocaine (5 mg/kg ideal bodyweight) was administered over 1.5 h at an average rate of 50 μg/kg per minute under the monitoring. If any serious adverse events occurred, the IV lidocaine infusion was immediately discontinued and the participant was managed appropriately.

### Assessments

Basic information was recorded, along with medical and organ transplant surgical history, neurological examination, laboratory tests of liver and kidney function, and current list of pain medications. Pain intensity and patient’s quality of life (QoL) were evaluated by NRS and Brief Pain Inventory (BPI), and prior and post-lidocaine infusion, all patients were followed-up routinely for 6 months. Potential adverse reactions related to IV lidocaine including nausea, vomiting, dizziness, arrhythmia, hallucinations, tremor, and hypotension were recorded for safety assessments. To assess the immediate and long-term effects of lidocaine infusion on systemic status and organ functions, blood routine, liver, and renal function laboratory tests were obtained on the second day and 2 weeks after the infusion and compared with baseline values.

### Case presentation

A total of five transplant recipients suffering from intractable zoster-associated neuralgia visited our center from April 2019 to April 2024. Information was extracted from medical records, and the pathophysiological characteristics and therapeutic management of the patients are described in Table 1. These patients suffered from severe spontaneous and intermittent pain, characterized by burning, throbbing, and tingling, with a NRS score of 8–9. Lidocaine patches and systemic analgesics of pregabalin, tramadol, oxycodone–acetaminophen, and fentanyl transdermal patch were used according to guideline recommendation (Werner et al., 2017). To reduce the use of heavy analgesic regimens, which have potential toxicities for transplanted organ function, interventional procedures such as CT-guided dorsal root ganglion pulsed radiofrequency, radiofrequency thermocoagulation, or short-term spinal cord stimulation have been previously performed. The patients still complained about moderate to severe pain, with sleep disturbance, depressive status, and worsened QoL. As a poor response to conservative and procedural management, we planned the intravenous lidocaine infusion as a complementary therapy.

**TABLE 1:** Patient characteristics and treatments.

Case	Gender	Age	Dermatome involved	Duration of pain (m)	Type of transplantation	Pharmacological treatment	Previous neuromodulation treatment
1	M	49	T11-12	6	Kidney	PGB 300 mg/day + FTP 50 μg/h	PRF
2	M	55	T6-8	1	Kidney	PGB 300 mg/day + OAT 3#/day + tramadol 200 mg/day	PRT
3	M	56	C7-8	2	Kidney	PGB 150 mg/day + tramadol 200 mg/day	st-SCS
4	M	59	T5-6	0.8	Lung	PGB 300 mg/day + OAT 4#/day	PRF
5	M	45	T1-2	3	Kidney	PGB 300 mg/day + tramadol 200 mg/day	PRF

PGB, pregabalin; FTP, fentanyl transdermal patch; OAT, 5 mg oxycodone and 325 mg acetaminophen; PRF, pulsed radiofrequency; RFT, radiofrequency thermocoagulation; st-SCS, short-term spinal cord stimulation.

Patients 1, 3, and 5 received IV lidocaine infusions once a week for 3 consecutive weeks. Due to transportation barriers, patients 2 and 4 missed one instance of IV lidocaine infusions. The patients experienced sound pain relief after the intravenous infusion therapy. Table 2 illustrates the changes of NRS scores in individuals. All patients exhibited a reduction in pain intensity, as evidenced by a decrease in the average NRS score from 8.4 to 4.2 (post-first lidocaine infusion) and 3.2 (post-second lidocaine infusion). At 6 months, the pain was bearable, and patients 2, 3, and 5 were free of oral medication. Patients 1 and 4 exhibited a reduction in analgesic requirements, with a daily dose of 150 mg of pregabalin, and therefore experienced slight pain recurrence. The BPI-QoL assesses general activity, mood, walking ability, normal work, relations with other people, sleep, and enjoyment of life. Table 3 demonstrates that the BPI-QoL score significantly decreased after the IV lidocaine treatment at all time points. Regarding patient satisfaction, patients 1, 2, and 5 reported their treatment satisfaction as “excellent.”

**TABLE 2:** Pain scores.

Case	NRS						
	Before IV	Post 1st IV	Post 2nd IV	Post 3rd IV	1 month	3 months	6 months
1	9	5	3	2	2	3	4
2	8	5	4	-	2	3	2
3	8	3	3	3	3	2	3
4	9	5	3	-	2	2	4
5	8	3	3	2	1	3	2

**TABLE 3:** BPI-QoL and patient satisfaction.

Case	BPI-QoL				Patient satisfaction
	Before IV	1 month	3 months	6 months	
1	42	18	12	10	Excellent
2	37	19	7	5	Excellent
3	45	15	11	8	Good
4	38	13	9	9	Very good
5	40	13	10	6	Excellent

Normal blood test results showed no major changes in typical liver and renal function post-lidocaine infusion. Patient 3 experienced short and mild numbness in the mouth and dizziness after the first IV lidocaine therapy, but no severe adverse effects were reported.

## Discussion

Neurological complications are frequent in SOT recipients and may largely contribute to morbidity and mortality. Previous studies reported the incidence of HZ varied between 3.5% and 16.2%, and the incidence of PHN ranges from 10% to 50% of HZ cases in transplant recipients (Koo et al., 2014; Pavlopoulou et al., 2015; Kho et al., 2021). This chronic painful disease negatively impacts the QoL and becomes more intractable as the disease progresses; therefore, the multimodal approach for pain and management is an urgent need. Medical management for zoster-associated neuralgia includes antiepileptic drugs (gabapentin or pregabalin), tricyclic antidepressants (amitriptyline), selective norepinephrine reuptake inhibitors (duloxetine), and local analgesics (5% lidocaine patches) as first-line drugs; opioids, 8% topical capsaicin cream as second- or third-line treatment (Moulin et al., 2014). Patients after solid organ transplantation are at risk for high toxicity, and immunosuppression therapies may cause indirect and drug-related adverse effects; thus, attention should be paid when chosen analgesic from the above. In general, heavy analgesic regimens and long-term medication should be avoided, if possible. It is important to monitor liver and kidney function carefully during high-dose analgesia. The measurement of blood urea nitrogen and creatinine levels, urine albumin, and the calculation of the creatinine clearance are useful for the evaluation of renal insufficiency and the adjustment of drug dosing. Another problem in transplant patients is that of drug–drug interactions (DDIs) with immunosuppressive drugs. Calcineurin inhibitors (CNIs) such as cyclosporine, tacrolimus, and pimecrolimus should be avoided with NSAIDs due to the risk of kidney injury. The antiepileptic drugs (e.g., phenytoin and carbamazepine) may interact with CNIs, and careful monitoring of CNI blood levels is therefore necessary (Vanhove et al., 2017; Lemke et al., 2023). Maintaining vigilance for an increased risk of adverse events, including respiratory depression, constipation, sedation, confusion, delirium, and cognitive impairment, is critical in SOT recipients (Herborn and Parulkar, 2017). In this case report, we examined five patients with creatinine clearance ranging from 30 to 60 mL/min. These patients were administered pregabalin at doses not exceeding 300 mg per day in combination with low-dose opioids. Notably, no significant adverse effects were observed in this cohort.

Systemic lidocaine used in continuous infusion has analgesic, antihyperalgesic, as well as anti-inflammatory properties (Soto et al., 2018; Castro et al., 2023). Intravenous lidocaine has a substantial effect on damaged neural tissues, blocks neuropathic pain via its action on sodium channels that reduce input from nociceptors, and blocks central hyperexcitability. Previous studies described the effectiveness of IV lidocaine for chronic pain and perioperative pain (Kim et al., 2018; Weibel et al., 2018). A meta-analysis of 15 studies found single lidocaine infusion for the treatment of neuropathic pain is effective in the immediate post-transfusion period, over 4 weeks, and it does not have a long-lasting effect (Zhu et al., 2019). In this case report, the patients received 2–3 times of lidocaine infusion treatment and reported sound pain relief with high satisfaction after 6-month follow-up. Furthermore, well-designed RCTs evaluating the effects of repeated lidocaine administration and long-lasting effects are required.

Common adverse effects of lidocaine injection may include slow heart rate, muscle twitching, seizures, respiratory depression, dizziness, nausea, and vomiting. No serious adverse events were observed in this study, and laboratory tests indicated no major changes. The higher dosage of lidocaine-produced plasma levels is associated with an increased risk of adverse effects. Previous studies reported that 1 mg/kg of lidocaine infusion was not superior to placebo, whereas 2 mg/kg is effective (Tremont-Lukats et al., 2006). Attal et al. used 5 mg/kg lidocaine at a high infusion rate of 167 μg/kg per minute for approximately 30 min, which leads to adverse cardiovascular events (Attal et al., 2004). The lidocaine infusion protocol was according to our previous study, which administered 5 mg/kg lidocaine, prolonged infusion over 1.5 h that reduced the incidence of cardiac, respiratory, and central nervous system complications mentioned above (Liu et al., 2018).

There are multiple limitations in this study, including data collection from a single center, small sample size, and short follow-up time. The results presented here should be regarded as preliminary data, and prospective randomized clinical trials with large sample sizes and long-term follow-up time are needed to validate these results. Conversely, patients in this study with a relatively good liver and renal function, classified as Child–Pugh B and chronic kidney disease stage 3, were assessed. Pain management in critically ill patients with compromised hepatic and renal function remains a significant challenge.

## Conclusion

In this case series at a tertiary care teaching hospital, our findings suggest that intravenous lidocaine provided effective pain relief, as a multimodal analgesic option for SOT patients with intractable zoster-associated neuralgia.

## Data Availability

The raw data supporting the conclusions of this article will be made available by the authors, without undue reservation.
